# Supplementary material for: Associations between job demands, job resources and patient-related burnout among physicians: results from a multicentre observational study
Source: BMJ Open. 2020 Sep 24;10(9):e038466. doi: 10.1136/bmjopen-2020-038466 (PMC7517563; doi:10.1136/bmjopen-2020-038466)
Supplement: Supplementary data [file bmjopen-2020-038466supp001.pdf]

Supplementary tables

Table A1. The job resources, job demands and patient-related burnout items and corresponding psychometric properties

Construct (Cronbach's α)	Subscale (Cronbach's α)	Mean (SD)*	Item code (corrected item-total correlation)	Factor loadings	Item
Job resources	Relationships with colleagues (α = 0.75)	3.43 (0.47)	(0.34)	0.35	Asking colleagues for support
			(0.62)	0.71	Good understanding with colleagues
			(0.54)	0.60	Conflicts with colleagues
			(0.65)	0.77	Pleasant atmosphere between colleagues
			(0.48)	0.56	Unpleasant events with colleagues
	Participation in decision-making (α = 0.82)	3.60 (0.74)	(0.63)	0.63	Participation in important decisions
			(0.73)	0.85	Participation in assignment of tasks
			(0.60)	0.68	Participation in timetable planning
			(0.61)	0.63	Influence on work
	Development oppurtunities (α = 0.84)	4.14 (0.69)	(0.68)	-0,82	Opportunity to learn new things in work
			(0.75)	-0.77	Opportunity to grow and develop
			(0.68)	-0.72	Opportunity to achieve something in work
	Leaders' inspiration (α = 0.91)	2.64 (0.82)	(0.83)	0.87	Enthusiasm of the leader
			(0.78)	0.83	Good example of the leader
			(0.82)	0.85	Ratification of the leader
			(0.79)	0.81	Clear vision of the leader
	Relationships with patients (α = 0.71)	3.81 (0.62)	(0.33)	0.29	I find my present clinical work personally rewarding
			(0.61)	0.72	I feel a strong personal connection with my patients
			(0.57)	0.74	The gratitude displayed by my patients keeps me going
			(0.51)	0.64	I am having a positive impact on a socio-economically disadvantaged population
Job demands	Bureaucratic load (α = 0.74)	3.11 (0.73)	(0.52)	0.60	Not burdensome to burdensome policies and procedures

			(0.58)	0.72	Necessary to unnecessary policies and procedures
			(0.62)	0.80	Effective to ineffective policies and procedures
	Workload (α = 0.79)	2.85 (0.52)	(0.39)	0.47	Amount of work
			(0.58)	0.69	Extra work to complete tasks
			(0.61)	0.70	Hurry in work
			(0.56)	0.64	Arrear in work
			(0.54)	0.59	Problems with work pace
			(0.60)	0.65	Problems with workload
Patient-related burnout	Patient-related burnout (α = 0.83)	1.91 (0.63)	(0.65)	0.81	Do you find it hard to work with patients?
			(0.67)	0.69	Do you find it frustrating to work with patients?
			(0.72)	0.56	Does it drain your energy to work with patients?
			(0.61)	0.50	Do you feel that you give more than you get back when you work with patients?
			(0.54)	0.74	Are you tired of working with patients?
			(0.46)	0.75	Do you sometimes wonder how long you will be able to continue working with patients?

*SD = standard deviation

Table A2. Inter-scale correlations for the subscales of the job resources and job demands constructs

	Job resources					Job demands	
	Relationships with colleagues	Participation in decision-making	Development possibilities	Inspiration by the leader	Relationships with patients	Bureaucratic load	Workload
Relationships with colleagues	1	0.32**	0.30**	0.34**	0.15**	-	-
Participation in decision making	-	1	0.40**	0.38**	0.17**	-	-
Development oppurtunities	-	-	1	0.34**	0.28**	-	-
Leaders' inspiration	-	-	-	1	0.10*	-	-
Relationships with patients	-	-	-	-	1	-	-
Bureaucratic load	-	-	-	-	-	1	0.15**
Workload	-	-	-	-	-	-	1

*significant Pearson correlation p<0.01
** significant Pearson correlation p<0.05
