# Supplementary material for: Associations between job demands, job resources and patient-related burnout among physicians: results from a multicentre observational study
Source: BMJ Open. 2020 Sep 24;10(9):e038466. doi: 10.1136/bmjopen-2020-038466 (PMC7517563; doi:10.1136/bmjopen-2020-038466)
Supplement: Author's manuscript [file bmjopen-2020-038466.draft_revisions.pdf]

BMJ Open is committed to open peer review. As part of this commitment we make the peer review history of every article we publish publicly available.

When an article is published we post the peer reviewers' comments and the authors' responses online. We also post the versions of the paper that were used during peer review. These are the versions that the peer review comments apply to.

The versions of the paper that follow are the versions that were submitted during the peer review process. They are not the versions of record or the final published versions. They should not be cited or distributed as the published version of this manuscript.

BMJ Open is an open access journal and the full, final, typeset and author-corrected version of record of the manuscript is available on our site with no access controls, subscription charges or pay-per-view fees (http://bmjopen.bmj.com).

If you have any questions on BMJ Open's open peer review process please email
info.bmjopen@bmj.com

BMJ Open

The associations between job demands, job resources and patient-related burnout among doctors: results from a multicentre observational study

Journal:	*BMJ Open*
Manuscript ID	bmjopen-2020-038466
Article Type:	Original research
Date Submitted by the Author:	12-Mar-2020
Complete List of Authors:	Scheepers, Renee; Erasmus University Rotterdam, Erasmus School of Health Policy and Management<br>Silkens, Milou; University College London, Research Department of Medical Education<br>van den Berg, Joost; Amsterdam University Medical Centres, Internal Medicine<br>Lombarts, Kiki; Amsterdam University Medical Centres, Medical Psychology
Keywords:	Health policy < HEALTH SERVICES ADMINISTRATION & MANAGEMENT, Human resource management < HEALTH SERVICES ADMINISTRATION & MANAGEMENT, Quality in health care < HEALTH SERVICES ADMINISTRATION & MANAGEMENT, MEDICAL EDUCATION & TRAINING

SCHOLARONE™
Manuscripts

I, the Submitting Author has the right to grant and does grant on behalf of all authors of the Work (as defined in the below author licence), an exclusive licence and/or a non-exclusive licence for contributions from authors who are: i) UK Crown employees; ii) where BMJ has agreed a CC-BY licence shall apply, and/or iii) in accordance with the terms applicable for US Federal Government officers or employees acting as part of their official duties; on a worldwide, perpetual, irrevocable, royalty-free basis to BMJ Publishing Group Ltd ("BMJ") its licensees and where the relevant Journal is co-owned by BMJ to the co-owners of the Journal, to publish the Work in this journal and any other BMJ products and to exploit all rights, as set out in our licence.

The Submitting Author accepts and understands that any supply made under these terms is made by BMJ to the Submitting Author unless you are acting as an employee on behalf of your employer or a postgraduate student of an affiliated institution which is paying any applicable article publishing charge ("APC") for Open Access articles. Where the Submitting Author wishes to make the Work available on an Open Access basis (and intends to pay the relevant APC), the terms of reuse of such Open Access shall be governed by a Creative Commons licence – details of these licences and which Creative Commons licence will apply to this Work are set out in our licence referred to above.

Other than as permitted in any relevant BMJ Author's Self Archiving Policies, I confirm this Work has not been accepted for publication elsewhere, is not being considered for publication elsewhere and does not duplicate material already published. I confirm all authors consent to publication of this Work and authorise the granting of this licence.

For peer review only

The associations between job demands, job resources and patient-related burnout among doctors: results from a multicentre observational study

Renée A. Scheepers, M.Sc., Ph.D.[1,2]

Milou E.W.M. Silkens, M.Sc., Ph.D.[2,3]

Joost W. van den Berg, MD, Ph.D.[2]

Kiki M.J.M.H. Lombarts, M.Sc., Ph.D., Professor[2]

[1]Research group Socio-Medical Sciences, Erasmus School of Health Policy and Management, Erasmus University of Rotterdam, The Netherlands

[2]Professional Performance & Compassionate Care Research Group, Department of Medical Psychology, Amsterdam University Medical Center, University of Amsterdam, Amsterdam, The Netherlands

[3]Research Department of Medical Education, UCL Medical School, University College London, London, The United Kingdom

Corresponding author: Renée Scheepers

Research Group Socio-Medical Sciences, Erasmus School of Health Policy and Management, Erasmus University of Rotterdam, The Netherlands.

Burgemeester Oudlaan 50, PO Box 1738

3062 PA Rotterdam, The Netherlands

Telephone: +31 10 4020554

Fax: +31 10 4020554

Email: scheepers@eshpm.eur.nl

For peer review only - http://bmjopen.bmj.com/site/about/guidelines.xhtml

ABSTRACT

Objectives To investigate associations of job demands and resources with patient-related burnout among doctors.

Design Multicentre observational study.

Setting Fifty medical departments at 14 (academic and non-academic) hospitals in the Netherlands.

Participants Four hundred sixty-five doctors (81.2% response rate), comprising 385 (82.8%) medical specialists and 80 (17.2%) residents.

Main outcome measures Job demands (workload and bureaucratic load), job resources (participation in decision making, development opportunities, leader's inspiration, relationships with colleagues and patients) – measured with the validated Questionnaire of Experience and Evaluation of Work and Physician Worklife Survey – and patient-related burnout, measured using the validated Copenhagen Burnout Inventory.

Results Patient-related burnout was associated positively with workload (b = 0.36; 95% confidence interval (CI), 0.25 to 0.48; p < 0.001) and negatively with development opportunities (b = –0.18; 95% CI, –0.27 to –0.08; p < 0.001) and relationships with patients (b = –0.12; 95% CI, –0.22 to –0.03; p = 0.01). Relationships with patients moderated the association between the bureaucratic load and patient related-burnout (b = –0.15; 95% CI, –0.27 to –0.04; p = 0.01).

Conclusions Doctors with high workloads and few development opportunities reported higher levels of patient-related burnout. Those with positive patient relationships were less likely to experience patient-related burnout, even in the presence of high bureaucratic loads. Health care organisations could consider proactive support of positive doctor–patient relationships to reduce the likelihood of doctors' patient-related burnout.

For peer review only - http://bmjopen.bmj.com/site/about/guidelines.xhtml

For peer review only

Article summary

Strengths and limitations of the study

- This study addressed a knowledge gap on how job demands and job resources are associated with patient-related burnout among doctors.
- Job demands, job resources and patient-related burnout were measured by validated instruments that were selected based on both a needs assessment among practicing doctors and the evidence-based job demands and resources model.
- This multicentre study was conducted in academic and non-academic medical centres and included multiple specialties, to warrant generalizability of findings to diverse hospital-based settings.
- Our study resulted in a substantial response rate (71.3%) and accounted for potential confounders, though our findings may have been affected by self-selection of participants.
- The cross-sectional study design precluded the assessment of causal associations, yet our cross-sectional findings did align with longitudinal findings of related research.

For peer review only

Funding

The study was funded by the Dutch Ministry of Social Affairs and Employment. This Ministry had no role in the study design; in the collection, analysis, and interpretation of data; in the writing of the report; and in the decision to submit the article for publication. All authors confirm their independence from funders and had full access to all of study data and take responsibility for the integrity of the data and the accuracy of the data analysis is also required.

Ethical approval

Ethical approval was waived by the Medical Ethics Committee of the Amsterdam University Medical Centre (ID XT4-118). All participants gave informed consent before taking part.

Conflict of interest

The authors report no support from any organisation for the submitted work; no financial relationships with any organisations that might have an interest in the submitted work in the previous three years, no other relationships or activities that could appear to have influenced the submitted work.

License

The Corresponding Author does grant on behalf of all authors, a worldwide license to the Publishers and its licensees in perpetuity, in all forms, formats and media (whether known now or created in the future), to i) publish, reproduce, distribute, display and store the Contribution, ii) translate the Contribution into other languages, create adaptations, reprints, include within collections and create summaries, extracts and/or, abstracts of the Contribution and convert or allow conversion into any format including without limitation audio, iii) create any other derivative work(s) based in whole or part on the on the Contribution, iv) to exploit all subsidiary rights to exploit all subsidiary rights that currently exist or as may exist in the future in the Contribution, v) the inclusion of electronic links from the Contribution to third party material where-ever it may be located; and, vi) licence any third party to do any or all of the above.

For peer review only - http://bmjopen.bmj.com/site/about/guidelines.xhtml

For peer review only

Author contributorship

RS conceptualized and designed the study; acquired and interpreted study data; drafted the article; gave final approval of the version to be published; and agrees to be accountable for all aspects of the study in ensuring that questions related to the accuracy or integrity of any part of the study are appropriately investigated and resolved.

MS contributed to the design of the study; analyzed and interpreted study data; drafted the article; gave final approval of the version to be published; and agrees to be accountable for all aspects of the study in ensuring that questions related to the accuracy or integrity of any part of the study are appropriately investigated and resolved.

JB contributed to the design of the study; interpreted study data; critically revised the article; gave final approval of the version to be published; and agrees to be accountable for all aspects of the study in ensuring that questions related to the accuracy or integrity of any part of the study are appropriately investigated and resolved.

ML conceptualized and designed the study; acquired and interpreted study data; critically revised the article; gave final approval of the version to be published; and agrees to be accountable for all aspects of the study in ensuring that questions related to the accuracy or integrity of any part of the study are appropriately investigated and resolved.

For peer review only

Data sharing statement

No additional data available, as data are protected under contract with participating medical centers. Nonetheless, inquiries about potential research collaboration can be directed to Professor Kiki Lombarts (m.j.lombarts@amsterdamumc.nl).

Word count

2546 words

For peer review only - http://bmjopen.bmj.com/site/about/guidelines.xhtml

INTRODUCTION

In the last decade, research has revealed risks of doctor burnout to patient care quality.[1-3] Patient care quality is more likely to be suboptimal – as evidenced by increased numbers of safety incidents and lower levels of patient satisfaction – when doctors are burned out.[1-3] Doctor burnout rates are high worldwide, and this situation has been recognised as a global system-level problem.[4-8] Across health care systems, doctor burnout has been related to the stressful working conditions of modern medical practice, which involve heavy workloads in combination with constant time pressure and an excessive administrative burden.[9-11] These conditions sap doctors' energy by reducing autonomy and by limiting doctors' time for and attention to patients.[12 13] Connecting with patients – the very essence of being a doctor – has become increasingly challenging in modern practice.

This connection with patients and the provision of patient care used to be the main source of doctors' professional satisfaction and sense of meaning in work.[13] Recently, however, doctors have reported exhaustion in providing patient care.[14 15] The degree of exhaustion that doctors relate to working with patients indicates patient-related burnout,[16] which is reported by 8% of specialists and 23% of residents.[15 17] Doctors report higher levels of patient-related burnout when exposed to work environments with higher quantitative demands.[15 18] In general, burnout is more likely to develop when job demands – stressful aspects of work (e.g. workloads) – are high. On the other hand, burnout may be minimised or prevented by the provision of job resources – energising aspects of work (e.g. development opportunities).[19 20]

Insight into which job demands and resources are related to patient-related burnout among doctors, however, remains limited; only two studies on this topic have been conducted to date.[15 18] These studies have not involved the examination of job demands and resources that are specifically relevant to medical practice (e.g. bureaucratic loads and relationships with patients). Moreover, these studies have not yet clarified how job demands and resources interact in relation to patient-related burnout; this interaction may matter in the context of burnout prevention as job demands less likely result in burnout when job resources are high.[19] To aid the targeting of

For peer review only - http://bmjopen.bmj.com/site/about/guidelines.xhtml

relevant components, the current study was conducted to investigate associations of job demands and resources to doctors' patient-related burnout, and to clarify the interaction of demands and resources in this context.

METHODS

Study population and setting

This study was part of a nationwide programme involving the measurement of perceived working conditions and well-being of medical staff in 50 departments at 14 hospitals in the Netherlands from April 2017 to June 2018. With an email describing the programme, we invited 572 doctors (468 medical specialists and 104 residents) to complete an online survey. Participation was voluntary, and participants' anonymity and confidentiality were safeguarded. The Medical Ethics Committee of the Amsterdam University Medical Centre waived the ethics approval requirement for this study.

Patient and public involvement

There were no patients involved in the study. Doctors were involved in the design of the study based on the needs assessment described below. The findings have been disseminated through oral presentations at conferences for doctors and through newsletters on the website of the Professional Performance and Compassionate Care Research Group (https://professionalperformance-amsterdam.com/en/). This platform also made the measurement tools on working conditions and well-being publicly available to hospitals.

Measures

The survey included questions about job demands, job resources and patient-related burnout. As job demands and resources vary across professional settings, we selected those most relevant to doctors' practice. This selection was based on the validated job demands and resources model[20-

For peer review only - http://bmjopen.bmj.com/site/about/guidelines.xhtml

[22] and on a needs assessment, consisting of two focus groups and a web-based survey. The two focus groups included 24 participants in total (doctors and residents) and explored potentially relevant job resources and demands; the web-based survey was completed by 218 participants (doctors and residents), who assigned priority to the most relevant job demands and resources in medical practice. The results of the focus groups and survey were discussed in the research team, leading to the ultimate selection of job demands and resources (see below).

Job demands included workload and bureaucratic load. Workload was measured using the validated six-item Questionnaire on the Experience and Evaluation of Work (QEEW), with responses structured by a four-point scale ranging from 1 ('never') to 4 ('always').[23] Bureaucratic load was measured by the validated Three-Item Red Tape Scale, consisting of one item (''How would you describe policies and procedures in your work division between the following opposite characteristics?'), with responses given on five-point scales ranging from 'not burdensome' to 'burdensome', 'necessary' to 'unnecessary', and 'effective' to 'ineffective'.[24]

Job resources included participation in decision making, development opportunities, leaders' inspiration, relationships with colleagues and relationships with patients. The first four resources were measured using the QEEW[23] and the fifth was measured using the validated Physician Worklife Survey.[25] Responses to the three items on relationships with colleagues and four items on the leader's inspiration were structured by a four-point scale ranging from 1 ('never') to 4 ('always'). Those to the four items each on participation in decision making, development opportunities, and relationships with patients were given on a five-point scale ranging from 1 ('totally disagree') to 5 ('totally agree'). As the original relationships with patients subscale had not been validated in Dutch, two researchers independently translated the English version into Dutch and agreed on the Dutch version, which another bilingual researcher back-translated. We resolved minor differences between the back translation and original, adjusting the forward translation to create the final Dutch version of the subscale.

For peer review only

Patient-related burnout was measured using the validated six-item Copenhagen Burnout Inventory, with responses structured by a five-point scale ranging from 1 ('totally disagree') to 5 ('totally agree').[16]

The survey also included questions about doctors' characteristics: training level (specialist/resident), years of experience, specialty (surgical/medical), type of employment (full time/part time) and sex (male/female). These data were included in the statistical analysis to adjust for potential confounding of associations of job resources and demands with patient-related burnout.

Statistical analysis

Sample characteristics were represented using frequencies and descriptive statistics. The psychometric properties of the job demands, job resources and patient-related burnout constructs were assessed using exploratory factor analysis (EFA) and reliability analysis (supplementary tables A1 and A2). For the EFA, we performed principal axis factoring with oblique rotation and chose the Kaiser–Guttman criterion and fixed factor models for extraction of the optimal number of factors.[26] The research team discussed the theoretical relevance of two job resources items with loadings < 0.40, and decided to retain them. The EFA yielded two job demands subscales (9 items), five job resources subscales (20 items) and one patient-related burnout subscale (6 items; supplementary table A1). Reliability was assessed according to internal consistency (satisfactory when Cronbach's $\alpha>0.70$[27]), inter-scale correlations (satisfactory when Pearson's $r<0.70$), and the item-total correlations (satisfactory when Pearson's $r>0.30$). Following the establishment of construct validity and reliability, mean subscale and total scores were calculated for each construct.

To assess the associations of job demands and resources with doctors' patient-related burnout, we used unadjusted and adjusted random-intercept generalised linear mixed models. These models allowed us to account for the hierarchical clustering of individuals within clinical departments within hospitals. In the unadjusted models, total mean scores (model 1) and

For peer review only - http://bmjopen.bmj.com/site/about/guidelines.xhtml

individual subscale scores (model 2) for the job demands and resources constructs served as explanatory variables, and scores on patient-related burnout served as the outcome variable. Subsequent models were adjusted for doctors' sex, post–MD degree years of experience, employment type (full-time/part-time), and type of respondent (medical specialist/resident). These analyses were performed using SPSS Statistics version 25 (IBM Corp., NY). Furthermore, we analyzed interactions between job demands and resources in relation to patient-related burnout by employing moderation analysis, using the SPSS macro PROCESS.[28] All results were reported using regression coefficients (b), their 95% confidence interval (95% CI), and p values (< 0.05).

RESULTS

In total, 465 doctors (82.8% specialists, 17.2% residents) from 50 clinical departments at 16 hospitals completed the questionnaire (81.2% response rate; table 1). Of them, 111 (23.9%) doctors originated from academic hospitals.

The job demands, job resources, and patient-related burnout subscales showed satisfactory to good internal consistency, and inter-scale as well as item-to-total correlations (supplementary tables A1 and A2). The unadjusted model 1 revealed significant associations of job demands and resources with patient-related burnout, confirmed by the adjusted model 2 (table 2). The unadjusted model 2 showed that patient-related burnout was associated significantly with the two job demands subscales (workloads and bureaucratic loads and the job resources subscales of development opportunities and relationships with patients. The job resources subscales of relationships with colleagues, participation in decision making, and leaders' inspiration were not associated with patient-related burnout (table 2). All of these associations except that with bureaucratic load were confirmed by the adjusted model (table 2).

Relationships with patients significantly moderated the relationship between bureaucratic load and patient related-burnout ($b_{interactionterm}$ = −0.15; 95% CI, −0.27 to −0.04; p = 0.01). Low and moderate, but not high, relationships with patients scores were associated with a significant

For peer review only - http://bmjopen.bmj.com/site/about/guidelines.xhtml

positive relationship between bureaucratic load and patient-related burnout (b_{low} = 0.21; 95% CI, 0.11 to 0.31; t = 4.18; p < 0.001 and $b_{moderate}$ = 0.10; 95% CI, 0.02 to 0.18; t = 2.35; p = 0.02).

DISCUSSION

Principal findings

The findings of this study suggest that doctors with high workloads and few development opportunities report higher levels of patient-related burnout. Those with positive patient relationships are less likely to experience such burnout, even in the presence of high bureaucratic loads.

Strengths and limitations of the study

This multicentre study used widely validated instruments to examine associations between job demands, job resources and patient-related burnout, which were selected based on needs assessment of practicing doctors' needs in alignment with the theoretical assumptions of the job demands and resources model. However, other job demands and resources also may be relevant, especially in other settings (e.g. non-Dutch systems and primary care). In our hospital setting, we achieved a high response rate from doctors in multiple specialities, and the sex distribution of the sample was consistent with national data.[29] As study participation was voluntary, subscale scores may be subject to self-selection bias. However, the observed associations of job demands and resources with patient-related burnout – the main focus of the current study – are in agreement with related findings from diverse settings, as detailed below. Furthermore, the cross-sectional study design precluded the assessment of causal associations. Previous research has identified longitudinal associations of job demands and resources with burnout,[30 31] which should be clarified for patient-related burnout in particular.

For peer review only - http://bmjopen.bmj.com/site/about/guidelines.xhtml

Comparison with other studies

Our results align with previous findings that job demands are associated with patient-related burnout in general.[15 18] We additionally showed that levels of patient-related burnout were lower among doctors who experienced positive relationships with patients. Doctors perceived positive relationships with patients when they, for example, perceived gratitude from their patients, or felt a strong personal connection with patients. These resources of doctors' work showed to keep doctors going, even in the face of excessive demands.[32] Indeed, doctors experiencing positive relationships with patients reported lower levels of exhaustion (i.e. patient-related burnout), even when exposed to high demands in terms of heavy bureaucratic loads.

These findings align with those of other research based on the job demands and resources model, which showed that particular resources buffer the negative impact of demands on well-being.[19] Specifically, previous research showed life satisfaction or work engagement are less likely to be impaired by job demands when job resource levels are high.[32 33] In the case of patient-related burnout, the negative impact of bureaucratic load in particular may be buffered by positive relationships with patients. Bureaucratic loads represent the extent to which doctors perceive policies and procedures as burdensome, ineffective or unnecessary.[24] Bureaucratic load and the related concept of administrative burden have been shown to be associated with work-related burnout.[11]

We observed lower levels of patient-related burnout in the presence of ample development opportunities, consistent with previous research;[18] development opportunities have also been shown to benefit doctors' work engagement, another indicator of well-being.[34] Development opportunities stimulate doctors' senses of capability, mastery and skill, which may enhance their sense of clinical competence and prevent stress or exhaustion in the face of clinically or emotionally demanding situations in patient care.[35] Indeed, learning and professional updating in the context of continuous medical education (CME) have been associated with lower levels of stress and burnout.[35] Therefore, CME activities may be considered when aiming to address patient-related burnout in medical practice.

For peer review only - http://bmjopen.bmj.com/site/about/guidelines.xhtml

In this study, patient-related burnout was not affected by job resources involving participation in decision making, leaders' inspiration or supportive collegial relationships; consistently, previous research revealed no association between these resources and doctors' well-being (i.e. work engagement and work-related burnout).[34 36 37] The lack of association with collegial relationships is surprising, as collegial and peer support has shown the potential to help doctors deal with stress and threats to well-being.[32] Such support may not be cultivated fully in the professional medical context due to time limitations or doctors' personal barriers (e.g. apprehension about peers' views on their ability to cope).[38 39] Full cultivation of collegial support could contribute to a sense of reduced professional isolation and the reduction of doctors' exhaustion in providing patient care.[32 40] For example, collegial support could be fostered by debrief groups in which peers exchange stressful experiences and together reflect on coping with challenges of patient care, e.g. emotionally demanding patients.[41]

Implications for practice and research

Although doctors are generally at low risk of patient-related burnout, even low levels of such burnout may seriously threaten the sustainability of their practice – as manifested by low levels of commitment to work[18] and job satisfaction.[15] Thus, each patient-related burnout risk factor should be prevented or resolved, which could be facilitated by optimising the balance between job demands and resources. Hospitals could consider how to reduce demands that interfere with doctor–patient relationships, or invest in professional development programmes (e.g. CME) that facilitate doctors' learning and development of positive relationships with patients. Such relationship development could also be fostered by addressing system- and organisation-level barriers to doctors' delivery of compassionate care (e.g. inadequate time with patients, unsupportive leadership, inadequate support personnel and non-facilitative practice structures).[42] Furthermore, positive relationships with patients could be fostered by mindfulness-based communication programs; these programs enhanced doctors' dedicated and non-judgmental attention towards patients' thoughts and emotions, which showed to facilitate

For peer review only

empathy towards patients.[43-45] Such programmes have also been shown to promote doctors' self-compassion and self-care in stressful practice environments, and may thus contribute to doctors' well-being and the prevention of (patient-related) burnout.[45]

Efforts to prevent patient-related burnout should include the creation of a healthy workplace with consideration of doctors' and patients' input, characterised (according to the work life model) by reasonable workloads, control over the practice environment, stimulating rewards, a supportive community, fair treatment of staff and professional values.[7 46] Care delivery according to professional values may be complicated by doctor burnout, as burned-out doctors are twice as likely to exhibit low levels of professionalism (i.e. low adherence to treatment guidelines, lack of professional integrity and low levels of empathy).[1] However, the effects of patient-related burnout on doctors' professionalism remain unclear. Furthermore, burnout in general has been associated with suboptimal patient care quality (e.g. patient safety and satisfaction),[1-3] yet, this is unclear for patient-related burnout in particular and should be studied in future research.

For peer review only

For peer review only - http://bmjopen.bmj.com/site/about/guidelines.xhtml

References

1. Panagioti M, Geraghty K, Johnson J, et al. Association between physician burnout and patient safety, professionalism, and patient satisfaction: a systematic review and meta-analysis. *JAMA Int Med* 2018;178(10):1317-30.
2. Hall LH, Johnson J, Watt I, et al. Healthcare staff wellbeing, burnout, and patient safety: A systematic review. *PLoS One* 2016;11(7):e0159015.
3. Salyers MP, Bonfils KA, Luther L, et al. The Relationship Between Professional Burnout and Quality and Safety in Healthcare: A Meta-Analysis. *J Gen Int Med* 2016:1-8.
4. Physician satisfaction and burnout at different career stages. *Mayo Clin Proc* 2013. Elsevier.
5. Shanafelt TD, Balch CM, Bechamps GJ, et al. Burnout and career satisfaction among American surgeons. *Ann Surg* 2009;250(3):463-71.
6. Prins JT, Gazendam-Donofrio SM, Tubben BJ, et al. Burnout in medical residents: a review. *Med Educ* 2007;41(8):788-800.
7. Montgomery A, Panagopoulou E, Esmail A, et al. Burnout in healthcare: the case for organisational change. *BMJ* 2019;366:l4774.
8. National Academies of Sciences E, Medicine. Taking action against clinician burnout: a systems approach to professional well-being: National Academies Press 2019.
9. West CP, Dyrbye LN, Erwin PJ, et al. Interventions to prevent and reduce physician burnout: a systematic review and meta-analysis. *Lancet* 2016
10. Panagioti M, Panagopoulou E, Bower P, et al. Controlled Interventions to Reduce Burnout in Physicians: A Systematic Review and Meta-analysis. *JAMA Int Med* 2016
11. Rao SK, Kimball AB, Lehrhoff SR, et al. The Impact of Administrative Burden on Academic Physicians: Results of a Hospital-Wide Physician Survey. *Acad Med* 2017;92(2):237-43. doi: 10.1097/acm.0000000000001461
12. Boerjan M, Bluyssen SJ, Bleichrodt RP, et al. Work-related health complaints in surgical residents and the influence of social support and job-related autonomy. *Med Educ* 2010;44(8):835-44.
13. Shanafelt TD. Enhancing meaning in work: A prescription for preventing physician burnout and promoting patient-centered care. *JAMA* 2009;302(12):1338-40. doi: 10.1001/jama.2009.1385
14. Chou L-P, Li C-Y, Hu SC. Job stress and burnout in hospital employees: comparisons of different medical professions in a regional hospital in Taiwan. *BMJ Open* 2014;4(2):e004185.
15. Wright JG, Khetani N, Stephens D. Burnout among faculty physicians in an academic health science centre. *Paediatrics & child health* 2011;16(7):409-13.
16. Kristensen TS, Borritz M, Villadsen E, et al. The Copenhagen Burnout Inventory: A new tool for the assessment of burnout. *Work & Stress* 2005;19(3):192-207.
17. Ratnakaran B, Prabhakaran A, Karunakaran V. Prevalence of burnout and its correlates among residents in a tertiary medical center in Kerala, India: A cross-sectional study. *J Postgr Med* 2016;62(3):157.
18. Ilić IM, Arandjelović MŽ, Jovanović JM, et al. Relationships of work-related psychosocial risks, stress, individual factors and burnout–Questionnaire survey among emergency physicians and nurses. *Med Pr* 2017;68(2):167-78.
19. Bakker AB, Hakanen JJ, Demerouti E, et al. Job resources boost work engagement, particularly when job demands are high. *J Educ Psych* 2007;99(2):274.
20. Bakker AB, Demerouti E. The job demands-resources model: State of the art. *J Manag Psych* 2007;22(3):309-28.
21. Teoh K, Hassard J, Cox T. Doctors' perceived working conditions and the quality of patient care: a systematic review. *Work & Stress* 2019;33(4):385-413. doi: 10.1080/02678373.2019.1598514
22. Bakker AB, Lieke L, Prins JT, et al. Applying the job demands–resources model to the work–home interface: A study among medical residents and their partners. *J Voc Beh* 2011;79(1):170-80.

For peer review only - http://bmjopen.bmj.com/site/about/guidelines.xhtml

23. Veldhoven Mv, Jonge Jd, Broersen S, et al. Specific relationships between psychosocial job conditions and job-related stress: A three-level analytic approach. *Work & Stress* 2002;16(3):207-28.
24. Borry EL. A new measure of red tape: Introducing the three-item red tape (TIRT) scale. *Int Publ Manag J* 2016;19(4):573-93.
25. Williams ES, Konrad TR, Linzer M, et al. Refining the measurement of physician job satisfaction: results from the Physician Worklife Survey. *Med Care* 1999:1140-54.
26. Schönrock-Adema J, Heijne-Penninga M, van Hell EA, et al. Necessary steps in factor analysis: enhancing validation studies of educational instruments. The PHEEM applied to clerks as an example. *Med Teach* 2009;31(6):e226-e32.
27. Cronbach LJ. Coefficient alpha and the internal structure of tests. *Psychometrika* 1951;16(3):297-334.
28. Hayes AF. Introduction to mediation, moderation, and conditional process analysis: A regression-based approach: Guilford Publications 2017.
29. Van der Meulen L. Aantal werkzame specialisten per specialisme en uitstroom van Specialisten in de komende 20 jaar: Capaciteitsorgaan, 2019.
30. Hakanen JJ, Schaufeli WB, Ahola K. The Job Demands-Resources model: A three-year cross-lagged study of burnout, depression, commitment, and work engagement. *Work & Stress* 2008;22(3):224-41. doi: 10.1080/02678370802379432
31. Wang Y, Huang J, You X. Personal resources influence job demands, resources, and burnout: A one-year, three-wave longitudinal study. *Soc Beh Pers Int J* 2016;44(2):247-58.
32. Wallace JE, Lemaire J. On physician well being—you'll get by with a little help from your friends. *Soc Sc Med* 2007;64(12):2565-77.
33. Hakanen JJ, Bakker AB, Demerouti E. How dentists cope with their job demands and stay engaged: the moderating role of job resources. *Eur J Oral Sc* 2005;113(6):479-87. doi: 10.1111/j.1600-0722.2005.00250.x
34. Scheepers RA, Lases LS, Arah OA, et al. Job Resources, Physician Work Engagement, and Patient Care Experience in an Academic Medical Setting. *Acad Med* 2017
35. Kushnir T, Cohen AH, Kitai E. Continuing medical education and primary physicians' job stress, burnout and dissatisfaction. *Med Educ* 2000;34(6):430-36. doi: 10.1046/j.1365-2923.2000.00538.x
36. Verweij H, van der Heijden FMMA, van Hooff MLM, et al. The contribution of work characteristics, home characteristics and gender to burnout in medical residents. *Adv Health Sc Educ* 2017;22(4):803-18. doi: 10.1007/s10459-016-9710-9
37. Hariharan TS, Griffin B. A review of the factors related to burnout at the early-career stage of medicine. *Med Teach* 2019;41(12):1380-91.
38. Wallace JE, Lemaire JB, Ghali WA. Physician wellness: a missing quality indicator. *Lancet* 2009;374(9702):1714-21.
39. Edwards N, Kornacki MJ, Silversin J. Unhappy doctors: what are the causes and what can be done? *BMJ* 2002;324(7341):835.
40. Shapiro J, Galowitz P. Peer support for clinicians: A programmatic approach. *Acad Med* 2016;91(9):1200-04.
41. Lightowlers SV, Thompson MK, Hunt SL. Not all interventions for burnout cost money: the value of debrief groups. *BMJ* 2017;358:j4377. doi: 10.1136/bmj.j4377
42. Rider EA, Gilligan MC, Osterberg LG, et al. Healthcare at the Crossroads: The Need to Shape an Organizational Culture of Humanistic Teaching and Practice. *J Gen Int Med* 2018;33(7):1092-99. doi: 10.1007/s11606-018-4470-2
43. Verweij H, van Ravesteijn H, van Hooff ML, et al. Does mindfulness training enhance the professional development of residents? A qualitative study. *Acad Med* 2018;93(9):1335-40.
44. Beckman HB, Wendland M, Mooney C, et al. The impact of a program in mindful communication on primary care physicians. *Acad Med* 2012;87(6):815-19.

For peer review only - http://bmjopen.bmj.com/site/about/guidelines.xhtml

45. Scheepers RA, Emke H, Epstein RM, et al. The impact of mindfulness-based interventions on doctors' well-being and performance: A systematic review. *Med Educ* 2020;54:138-149. doi: 10.1111/medu.14020
46. Leiter MP, Maslach C. Areas of worklife: A structured approach to organizational predictors of job burnout. Emotional and physiological processes and positive intervention strategies: Emerald Group Publishing Limited 2003:91-134.

For peer review only

For peer review only - http://bmjopen.bmj.com/site/about/guidelines.xhtml

Table 1. Characteristics of the study population

Characteristics	N (%)
Number of respondents	465 (100)
Male	222 (47.7)
Female	243 (52.3)
Type of respondent	
Medical specialist	385 (82.8)
Resident	80 (17.2)
Specialty	
Surgical	193 (41.5)
Non-surgical	226 (48.6)
Supporting	32 (6.9)
Non-medical	14 (3.0)
Years after completing M.D.	
0-5	40 (8.6)
6-10	93 (20.0)
11-15	88 (18.9)
16-21	80 (17.2)
22-45	161 (34.6)
46+	3 (0.6)
Type of contract	
Full-time	257 (55.3)
Part-time	208 (44.7)

For peer review only

For peer review only - http://bmjopen.bmj.com/site/about/guidelines.xhtml

Table 2. Unadjusted and adjusted models predicting the effect of job resources and job demands on patient related burnout by using total mean scores (model 1) or subscale scores (model 2)

		Unadjusted model	Adjusted model#
		Regression coefficient (95% CI; p-value)	Regression coefficient (95% CI; p-value)
Job resources	Total mean score	-1.15 (-0.28 to -0.01; 0.03)	-0.17 (-0.31 to -0.04; 0.01)
	Relationships with colleagues	0.09 (-0.04 to 0.22; 0.17)	0.08 (-0.06 to 0.21; 0.26)
	Participation in decision making	0.02 (-0.07 to 0.11; 0.73)	0.02 (-0.08 to 0.11; 0.72)
	Development opportunities	-0.17 (-0.26 to -0.07; 0.00)	-0.18 (-0.27 to -0.08; 0.00)
	Leaders' inspiration	0.04 (-0.04 to 0.12; 0.30)	0.03 (-0.05 to 0.11; 0.41)
	Relationships with patients	-0.12 (-0.22 to -0.03; 0.01)	-0.12 (-0.22 to -0.03; 0.01)
Job demands	Total mean score	0.29 (0.17 to 0.42; 0.00)	0.29 (0.17 to 0.42; 0.00)
	Workload	0.34 (0.23 to 0.45; 0.00)	0.36 (0.25 to 0.48; 0.00)
	Bureaucratic load	0.09 (0.01 to 0.17; 0.03)	0.08 (-0.00 to 0.16; 0.06)

#Adjusted for: doctors' sex, years of experience after obtaining an M.D., type of contract (full-time/part-time), and type of respondent (medical specialist/resident).

For peer review only

Supplementary tables

Table A1. The job resources, job demands and patient-related burnout items and corresponding psychometric properties

Construct (Cronbach's α)	Subscale (Cronbach's α)	Mean (SD)*	Item code (corrected item-total correlation)	Item
Job resources	Relationships with colleagues (α = 0.75)	3.43 (0.47)	(0.34)	Asking colleagues for support
			(0.62)	Good understanding with colleagues
			(0.54)	Conflicts with colleagues
			(0.65)	Pleasant atmosphere between colleagues
			(0.48)	Unpleasant events with colleagues
	Participation in decision-making (α = 0.82)	3.60 (0.74)	(0.63)	Participation in important decisions
			(0.73)	Participation in assignment of tasks
			(0.60)	Participation in timetable planning
			(0.61)	Influence on work
	Development oppurtunities (α = 0.84)	4.14 (0.69)	(0.68)	Opportunity to learn new things in work
			(0.75)	Opportunity to grow and develop
			(0.68)	Opportunity to achieve something in work
	Leaders' inspiration (α = 0.91)	2.64 (0.82)	(0.83)	Enthusiasm of the leader
			(0.78)	Good example of the leader
			(0.82)	Ratification of the leader
			(0.79)	Clear vision of the leader
	Relationships with patients (α = 0.71)	3.81 (0.62)	(0.33)	I find my present clinical work personally rewarding
			(0.61)	I feel a strong personal connection with my patients
			(0.57)	The gratitude displayed by my patients keeps me going
			(0.51)	I am having a positive impact on a socio-economically disadvantaged population
Job demands	Bureaucratic load (α = 0.74)	3.11 (0.73)	(0.52)	Not burdensome to burdensome policies and procedures
			(0.58)	Necessary to unnecessary policies and procedures
			(0.62)	Effective to ineffective policies and procedures
	Workload (α = 0.79)	2.85 (0.52)	(0.39)	Amount of work
			(0.58)	Extra work to complete tasks
			(0.61)	Hurry in work
			(0.56)	Arrear in work
			(0.54)	Problems with work pace
			(0.60)	Problems with workload
Patient-related burnout	Patient-related burnout (α = 0.83)	1.91 (0.63)	(0.65)	Do you find it hard to work with patients?

For peer review only - http://bmjopen.bmj.com/site/about/guidelines.xhtml

			(0.67)	Do you find it frustrating to work with patients?
			(0.72)	Does it drain your energy to work with patients?
			(0.61)	Do you feel that you give more than you get back when you work with patients?
			(0.54)	Are you tired of working with patients?
			(0.46)	Do you sometimes wonder how long you will be able to continue working with patients?

*SD = standard deviation

Table A2. Inter-scale correlations for the subscales of the job resources and job demands constructs

	**Job resources**					**Job demands**	
	Relationships with colleagues	Participation in decision-making	Development possibilities	Inspiration by the leader	Relationships with patients	Bureaucratic load	Workload
Relationships with colleagues	1	0.32**	0.30**	0.34**	0.15**	-	-
Participation in decision making	-	1	0.40**	0.38**	0.17**	-	-
Development oppurtunities	-	-	1	0.34**	0.28**	-	-
Leaders' inspiration	-	-	-	1	0.10*	-	-
Relationships with patients	-	-	-	-	1	-	-
Bureaucratic load	-	-	-	-	-	1	0.15**
Workload	-	-	-	-	-	-	1

*significant Pearson correlation p<0.01
** significant Pearson correlation p<0.05

For peer review only - http://bmjopen.bmj.com/site/about/guidelines.xhtml

STROBE Statement—checklist of items that should be included in reports of observational studies

	Item No	Recommendation	Page number
**Title and abstract**	1	(*a*) Indicate the study's design with a commonly used term in the title or the abstract	1
		(*b*) Provide in the abstract an informative and balanced summary of what was done and what was found	5
**Introduction**			
Background/rationale	2	Explain the scientific background and rationale for the investigation being reported	6-7
Objectives	3	State specific objectives, including any prespecified hypotheses	6-7
**Methods**			
Study design	4	Present key elements of study design early in the paper	7
Setting	5	Describe the setting, locations, and relevant dates, including periods of recruitment, exposure, follow-up, and data collection	7
Participants	6	(*a*) *Cohort study*—Give the eligibility criteria, and the sources and methods of selection of participants. Describe methods of follow-up<br>*Case-control study*—Give the eligibility criteria, and the sources and methods of case ascertainment and control selection. Give the rationale for the choice of cases and controls<br>*Cross-sectional study*—Give the eligibility criteria, and the sources and methods of selection of participants	7-8
		(*b*) *Cohort study*—For matched studies, give matching criteria and number of exposed and unexposed<br>*Case-control study*—For matched studies, give matching criteria and the number of controls per case	
Variables	7	Clearly define all outcomes, exposures, predictors, potential confounders, and effect modifiers. Give diagnostic criteria, if applicable	7-9
Data sources/ measurement	8*	For each variable of interest, give sources of data and details of methods of assessment (measurement). Describe comparability of assessment methods if there is more than one group	7-9
Bias	9	Describe any efforts to address potential sources of bias	9-10
Study size	10	Explain how the study size was arrived at	7
Quantitative variables	11	Explain how quantitative variables were handled in the analyses. If applicable, describe which groupings were chosen and why	9-10
Statistical methods	12	(*a*) Describe all statistical methods, including those used to control for confounding	9-10
		(*b*) Describe any methods used to examine subgroups and interactions	9-10
		(*c*) Explain how missing data were addressed	NA
		(*d*) *Cohort study*—If applicable, explain how loss to follow-up was addressed<br>*Case-control study*—If applicable, explain how matching of cases and controls was addressed<br>*Cross-sectional study*—If applicable, describe analytical methods taking account of sampling strategy	NA
		(*e*) Describe any sensitivity analyses	NA

For peer review only

For peer review only - http://bmjopen.bmj.com/site/about/guidelines.xhtml

Continued on next page

**Results**			
Participants	13*	(a) Report numbers of individuals at each stage of study—eg numbers potentially eligible, examined for eligibility, confirmed eligible, included in the study, completing follow-up, and analysed	10
		(b) Give reasons for non-participation at each stage	NA
		(c) Consider use of a flow diagram	NA
Descriptive data	14*	(a) Give characteristics of study participants (eg demographic, clinical, social) and information on exposures and potential confounders	Table 1
		(b) Indicate number of participants with missing data for each variable of interest	NA
		(c) *Cohort study*—Summarise follow-up time (eg, average and total amount)	
Outcome data	15*	*Cohort study*—Report numbers of outcome events or summary measures over time	
		Case-control study—Report numbers in each exposure category, or summary measures of exposure	
		Cross-sectional study—Report numbers of outcome events or summary measures	Table A1
Main results	16	(*a*) Give unadjusted estimates and, if applicable, confounder-adjusted estimates and their precision (eg, 95% confidence interval). Make clear which confounders were adjusted for and why they were included	Table 2
		(*b*) Report category boundaries when continuous variables were categorized	NA
		(*c*) If relevant, consider translating estimates of relative risk into absolute risk for a meaningful time period	NA
Other analyses	17	Report other analyses done—eg analyses of subgroups and interactions, and sensitivity analyses	10-11
**Discussion**			
Key results	18	Summarise key results with reference to study objectives	11
Limitations	19	Discuss limitations of the study, taking into account sources of potential bias or imprecision. Discuss both direction and magnitude of any potential bias	11
Interpretation	20	Give a cautious overall interpretation of results considering objectives, limitations, multiplicity of analyses, results from similar studies, and other relevant evidence	12-14
Generalisability	21	Discuss the generalisability (external validity) of the study results	11
**Other information**			
Funding	22	Give the source of funding and the role of the funders for the present study and, if applicable, for the original study on which the present article is based	2

*Give information separately for cases and controls in case-control studies and, if applicable, for exposed and unexposed groups in cohort and cross-sectional studies.

Note: An Explanation and Elaboration article discusses each checklist item and gives methodological background and published examples of transparent reporting. The STROBE checklist is best used in conjunction with this article (freely available on the Web sites of PLoS Medicine at http://www.plosmedicine.org/, Annals of Internal Medicine at http://www.annals.org/, and Epidemiology at http://www.epidem.com/). Information on the STROBE Initiative is available at www.strobe-statement.org.

For peer review only

BMJ Open

The associations between job demands, job resources and patient-related burnout among physicians: results from a multicentre observational study

Journal:	*BMJ Open*
Manuscript ID	bmjopen-2020-038466.R1
Article Type:	Original research
Date Submitted by the Author:	23-Jun-2020
Complete List of Authors:	Scheepers, Renee; Erasmus University Rotterdam, Erasmus School of Health Policy and Management<br>Silkens, Milou; University College London, Research Department of Medical Education<br>van den Berg, Joost; Amsterdam University Medical Centres, Internal Medicine<br>Lombarts, Kiki; Amsterdam University Medical Centres, Medical Psychology
**Primary Subject Heading**:	Mental health
Secondary Subject Heading:	Health services research, Medical education and training
Keywords:	Health policy < HEALTH SERVICES ADMINISTRATION & MANAGEMENT, Human resource management < HEALTH SERVICES ADMINISTRATION & MANAGEMENT, Quality in health care < HEALTH SERVICES ADMINISTRATION & MANAGEMENT, MEDICAL EDUCATION & TRAINING

SCHOLARONE™
Manuscripts

I, the Submitting Author has the right to grant and does grant on behalf of all authors of the Work (as defined in the below author licence), an exclusive licence and/or a non-exclusive licence for contributions from authors who are: i) UK Crown employees; ii) where BMJ has agreed a CC-BY licence shall apply, and/or iii) in accordance with the terms applicable for US Federal Government officers or employees acting as part of their official duties; on a worldwide, perpetual, irrevocable, royalty-free basis to BMJ Publishing Group Ltd ("BMJ") its licensees and where the relevant Journal is co-owned by BMJ to the co-owners of the Journal, to publish the Work in this journal and any other BMJ products and to exploit all rights, as set out in our licence.

The Submitting Author accepts and understands that any supply made under these terms is made by BMJ to the Submitting Author unless you are acting as an employee on behalf of your employer or a postgraduate student of an affiliated institution which is paying any applicable article publishing charge ("APC") for Open Access articles. Where the Submitting Author wishes to make the Work available on an Open Access basis (and intends to pay the relevant APC), the terms of reuse of such Open Access shall be governed by a Creative Commons licence – details of these licences and which Creative Commons licence will apply to this Work are set out in our licence referred to above.

Other than as permitted in any relevant BMJ Author's Self Archiving Policies, I confirm this Work has not been accepted for publication elsewhere, is not being considered for publication elsewhere and does not duplicate material already published. I confirm all authors consent to publication of this Work and authorise the granting of this licence.

For peer review only

The associations between job demands, job resources and patient-related burnout among physicians: results from a multicentre observational study

Renée A. Scheepers, M.Sc., Ph.D.1[1,2]

Milou E.W.M. Silkens, M.Sc., Ph.D.[2,3]

Joost W. van den Berg, MD, Ph.D.[2]

Kiki M.J.M.H. Lombarts, M.Sc., Ph.D., Professor[2]

[1]Research group Socio-Medical Sciences, Erasmus School of Health Policy and Management, Erasmus University of Rotterdam, The Netherlands

[2]Professional Performance & Compassionate Care Research Group, Department of Medical Psychology, Amsterdam University Medical Center, University of Amsterdam, Amsterdam, The Netherlands

[3]Research Department of Medical Education, UCL Medical School, University College London, London, The United Kingdom

Corresponding author: Renée Scheepers

Research Group Socio-Medical Sciences, Erasmus School of Health Policy and Management, Erasmus University of Rotterdam, The Netherlands.

Burgemeester Oudlaan 50, PO Box 1738

3062 PA Rotterdam, The Netherlands

Telephone: +31 10 4020554

Fax: +31 10 4020554

Email: scheepers@eshpm.eur.nl

For peer review only

ABSTRACT

Objectives To investigate associations of job demands and resources with patient-related burnout among physicians.

Design Multicentre observational study.

Setting Fifty medical departments at 14 (academic and non-academic) hospitals in the Netherlands.

Participants Four hundred sixty-five physicians (71.6% response rate), comprising 385 (82.8%) medical specialists and 80 (17.2%) residents.

Main outcome measures Job demands (workload and bureaucratic demands), job resources (participation in decision making, development opportunities, leader's inspiration, relationships with colleagues and patients) – measured with the validated Questionnaire of Experience and Evaluation of Work and Physician Worklife Survey – and patient-related burnout, measured using the validated Copenhagen Burnout Inventory.

Results Patient-related burnout was positively associated with workload (b = 0.36; 95% confidence interval (CI), 0.25 to 0.48; p < 0.001) and negatively associated with development opportunities (b = –0.18; 95% CI, –0.27 to –0.08; p < 0.001) and relationships with patients (b = –0.12; 95% CI, –0.22 to –0.03; p = 0.01). Relationships with patients moderated the association between bureaucratic demands and patient related-burnout (b = –0.15; 95% CI, –0.27 to –0.04; p = 0.01).

Conclusions Physicians with high workloads and few development opportunities reported higher levels of patient-related burnout. Those with positive patient relationships were less likely to experience patient-related burnout, even in the presence of excessive bureaucracy. Therefore, positive physician–patient relationships may be supported to reduce the likelihood of physicians' patient-related burnout. However, the specific support needed to effectively reduce patient-related burnout may vary per healthcare context and thus requires intensified research across health care systems and settings.

For peer review only

Article summary

Strengths and limitations of the study

- This study addressed a knowledge gap on how job demands and job resources are associated with patient-related burnout among physicians.
- Job demands, job resources and patient-related burnout were measured by validated instruments that were selected based on both a needs assessment among practicing physicians and the evidence-based job demands and resources model.
- This multicentre study was conducted in academic and non-academic medical centres and included multiple specialties, to warrant generalizability of findings to diverse hospital-based settings.
- Our study resulted in a substantial response rate (71.6%) and accounted for potential confounders, though our findings may have been affected by self-selection of participants.
- The cross-sectional study design precluded the assessment of causal associations, yet our cross-sectional findings did align with longitudinal findings of related research.

For peer review only

Funding

The study was funded by the Dutch Ministry of Social Affairs and Employment. This Ministry had no role in the study design; in the collection, analysis, and interpretation of data; in the writing of the report; and in the decision to submit the article for publication. All authors confirm their independence from funders and had full access to all of study data and take responsibility for the integrity of the data and the accuracy of the data analysis is also required.

Ethical approval

Ethical approval was waived by the Medical Ethics Committee of the Amsterdam University Medical Centre (ID XT4-118). All participants gave informed consent before taking part.

Competing interests

None declared.

License

The Corresponding Author does grant on behalf of all authors, a worldwide license to the Publishers and its licensees in perpetuity, in all forms, formats and media (whether known now or created in the future), to i) publish, reproduce, distribute, display and store the Contribution, ii) translate the Contribution into other languages, create adaptations, reprints, include within collections and create summaries, extracts and/or, abstracts of the Contribution and convert or allow conversion into any format including without limitation audio, iii) create any other derivative work(s) based in whole or part on the on the Contribution, iv) to exploit all subsidiary rights to exploit all subsidiary rights that currently exist or as may exist in the future in the Contribution, v) the inclusion of electronic links from the Contribution to third party material where-ever it may be located; and, vi) licence any third party to do any or all of the above.

For peer review only - http://bmjopen.bmj.com/site/about/guidelines.xhtml

Author contributorship

RS conceptualized and designed the study; acquired and interpreted study data; drafted the article; gave final approval of the version to be published; and agrees to be accountable for all aspects of the study in ensuring that questions related to the accuracy or integrity of any part of the study are appropriately investigated and resolved.

MS contributed to the design of the study; analyzed and interpreted study data; drafted the article; gave final approval of the version to be published; and agrees to be accountable for all aspects of the study in ensuring that questions related to the accuracy or integrity of any part of the study are appropriately investigated and resolved.

JB contributed to the design of the study; interpreted study data; critically revised the article; gave final approval of the version to be published; and agrees to be accountable for all aspects of the study in ensuring that questions related to the accuracy or integrity of any part of the study are appropriately investigated and resolved.

ML conceptualized and designed the study; acquired and interpreted study data; critically revised the article; gave final approval of the version to be published; and agrees to be accountable for all aspects of the study in ensuring that questions related to the accuracy or integrity of any part of the study are appropriately investigated and resolved.

Data sharing statement

No additional data available, as data are protected under contract with participating medical centers. Nonetheless, inquiries about potential research collaboration can be directed to Professor Kiki Lombarts (m.j.lombarts@amsterdamumc.nl).

Word count: 3083 words

For peer review only

INTRODUCTION

In the last decade, research has revealed risks of physician burnout to patient care quality.[1-3] Patient care quality is more likely to be suboptimal – as evidenced by increased numbers of safety incidents and lower levels of patient satisfaction – when physicians are burned out.[1-3] Physician burnout rates are high worldwide, and this situation has been recognised as a global system-level problem.[4-8] Across health care systems, physician burnout has been related to the stressful working conditions of modern medical practice, which involve heavy workloads in combination with constant time pressure and an excessive administrative burden.[9-11] These conditions sap physicians' energy by reducing autonomy and by limiting physicians' time for and attention to patients.[12 13] Connecting with patients – the very essence of being a physician – has become increasingly challenging in modern practice.

This connection with patients and the provision of patient care used to be the main source of physicians' professional satisfaction and sense of meaning in work.[13] Recently, however, physicians have reported exhaustion in providing patient care.[14 15] The degree of exhaustion that physicians relate to working with patients indicates patient-related burnout, while work-related burnout involves the exhaustion that professionals attribute to their work in general.[16] Physicians generally report higher levels of work-related than patient-related burnout.[15 17 18] However, even low levels of patient-related burnout can be problematic as they indicate physicians' exhaustion in their core task – caring for patients. Furthermore, patient-related burnout has been associated with absenteeism due to sickness.[19] Physicians report higher levels of patient-related burnout when exposed to work environments with higher quantitative demands.[15 20] In general, burnout is more likely to develop when job demands – stressful aspects of work (e.g. workloads) – are high. On the other hand, burnout may be minimised or prevented by the provision of job resources – energising aspects of work (e.g. development opportunities).[21-23]

For peer review only

Insight into which job demands and resources are related to physicians' patient-related burnout in particular , however, remains limited; few studies on this topic have been conducted to date.[15 16] These studies have not involved the examination of job demands and resources that are specifically relevant to medical practice (e.g. excessive bureaucracy and relationships with patients). Moreover, these studies have not yet clarified how job demands and resources interact in relation to patient-related burnout; this interaction may matter in the context of burnout prevention as job demands less likely result in burnout when job resources are high.[21] To aid the targeting of relevant components, the objective of the current study was to investigate associations of job demands and resources to physicians' patient-related burnout, and to clarify the interaction of demands and resources in this context.

METHODS

Study population and setting

This study investigated associations between job demands, job resources and patient-related burnout by conducting a nationwide programme involving a survey on perceived working conditions and well-being of medical staff in 50 departments at 14 hospitals in the Netherlands from April 2017 to June 2018. With an email describing the programme, we invited 649 physicians to complete an online survey. Participation was voluntary, and participants' anonymity and confidentiality were safeguarded. The Medical Ethics Committee of the Amsterdam University Medical Centre waived the ethics approval requirement for this study.

Patient and public involvement

There were no patients involved in the study. Physicians were involved in the design of the study based on the needs assessment described below. The findings have been disseminated through oral presentations at conferences for physicians and through newsletters on the website of the Professional Performance and Compassionate Care Research Group

(https://professionalperformance-amsterdam.com/en/). This platform also made the measurement tools on working conditions and well-being publicly available to hospitals.

Measures

The survey included validated questionnaires about job demands, job resources and patient-related burnout. As job demands and resources vary across professional settings, we selected those most relevant to physicians' practice. This selection was based on the validated job demands and resources model[22 24 25] and on a needs assessment, consisting of two focus groups and a web-based survey. The two focus groups included 24 participants in total (physicians and residents) and explored potentially relevant job resources and demands; the web-based survey was completed by 218 participants (physicians and residents), who assigned priority to the most relevant job demands and resources in medical practice. The results of the focus groups and survey were discussed in the research team, leading to the ultimate selection of job demands and resources (see below).

Job demands included workload and bureaucratic demands. Workload was measured using the validated six-item subscale on workload of the Questionnaire on the Experience and Evaluation of Work (QEEW), with responses structured by a four-point scale ranging from 1 ('never') to 4 ('always').[26] Bureaucratic demands were measured by the validated Three-Item Red Tape Scale, consisting of one item ("How would you describe policies and procedures in your work division between the following opposite characteristics?'), with responses given on five-point scales ranging from 'not burdensome' to 'burdensome', 'necessary' to 'unnecessary', and 'effective' to 'ineffective'.[27]

Job resources included participation in decision making, development opportunities, leaders' inspiration, relationships with colleagues and relationships with patients. The first four resources were measured using the QEEW[26] and the fifth was measured using the validated Physician Worklife Survey.[28] Responses to the three items on relationships with colleagues and four items on the leader's inspiration were structured by a four-point scale ranging from 1 ('never') to 4

For peer review only - http://bmjopen.bmj.com/site/about/guidelines.xhtml

('always'). Those to the four items each on participation in decision making, development opportunities, and relationships with patients were given on a five-point scale ranging from 1 ('totally disagree') to 5 ('totally agree'). As the original relationships with patients subscale had not been validated in Dutch, two researchers independently translated the English version into Dutch and agreed on the Dutch version, which another bilingual researcher back-translated. We resolved minor differences between the back translation and original, adjusting the forward translation to create the final Dutch version of the subscale.

Patient-related burnout was measured using the validated six-item Copenhagen Burnout Inventory, with responses structured by a five-point scale ranging from 1 ('totally disagree') to 5 ('totally agree').[16]

The survey also included questions about physicians' characteristics: training level (specialist/resident), years of experience, specialty (surgical/medical), type of employment (full time/part time) and sex (male/female). These data were included in the statistical analysis to adjust for potential confounding of associations of job resources and demands with patient-related burnout.

Statistical analysis

Sample characteristics were represented using frequencies and descriptive statistics. The psychometric properties of the job demands, job resources and patient-related burnout constructs were assessed using exploratory factor analysis (EFA) and reliability analysis (supplementary tables A1 and A2). For the EFA, we performed principal axis factoring with oblique rotation and chose the Kaiser–Guttman criterion and fixed factor models for extraction of the optimal number of factors.[29] The research team discussed the theoretical relevance of two job resources items with loadings < 0.40, and decided to retain them because they originated from validated questionnaires[26 28] and were considered to contribute meaningfully to the overall construct. The EFA yielded two job demands subscales (9 items), five job resources subscales (20 items) and one patient-related burnout subscale (6 items; supplementary table A1). Reliability

For peer review only - http://bmjopen.bmj.com/site/about/guidelines.xhtml

was assessed according to internal consistency (satisfactory when Cronbach's α>0.70[30]), inter-scale correlations (satisfactory when Pearson's r <0.70), and the item-total correlations (satisfactory when Pearson's r >0.30). Following the establishment of construct validity and reliability, mean subscale and total scores were calculated for each construct.

To assess the associations of job demands and resources with physicians' patient-related burnout, we used unadjusted and adjusted random-intercept generalised linear mixed models. These models allowed us to account for the hierarchical clustering of individuals within clinical departments within hospitals. In the unadjusted models, total mean scores (model 1) and individual subscale scores (model 2) for the job demands and resources constructs served as independent variables, and scores on patient-related burnout served as the dependent variable. Subsequent models were adjusted for physician characteristics, i.e. physicians' sex, post–MD degree years of experience, employment type (full-time/part-time), and type of respondent (medical specialist/resident). [31]

Furthermore, we analyzed interactions between job demands and resources in relation to patient-related burnout by employing moderation analysis, using the SPSS macro PROCESS.[31] Specifically, we conducted multiple regression analyses including the independent variables (i) a specific job demand (workload or bureaucratic demands), (ii) a specific job resource (participation in decision making, development opportunities, leaders' inspiration, relationships with colleagues or relationships with patients) and (iii) the interaction term of the respective job demand and resource, and (iv) patient-related burnout as the dependent variable. A significant interaction term indicated a moderation effect, which was inspected by performing simple slopes analysis to measure the conditional effects of the independent variable for three values of the moderator: (1) low score (-1SD), (2) the average score, and (3) high score (+1SD). All results were reported using regression coefficients (b), their 95% confidence interval (95% CI), and p values (< 0.05). All analyses were performed using SPSS Statistics version 25 (IBM Corp., NY).

For peer review only - http://bmjopen.bmj.com/site/about/guidelines.xhtml

RESULTS

In total, 465 physicians (82.8% specialists, 17.2% residents) from 50 clinical departments at 16 hospitals completed the questionnaire (71.6% response rate; table 1). Of them, 111 (23.9%) physicians originated from academic hospitals.

The job demands, job resources, and patient-related burnout subscales showed satisfactory to good internal consistency, and inter-scale as well as item-to-total correlations (supplementary tables A1 and A2). Our analyses of associations between job demands, job resources and patient-related burnout showed that the unadjusted model 1 revealed significant associations of job demands and resources with patient-related burnout, confirmed by the adjusted model 2 (table 2). The unadjusted model 2 showed that patient-related burnout was associated significantly with the two job demands subscales of workloads and bureaucracy and the job resources subscales of development opportunities and relationships with patients. The job resources subscales of relationships with colleagues, participation in decision making, and leaders' inspiration were not associated with patient-related burnout (table 2). All of these associations except that with the job demand subscale of bureaucracy were confirmed by the adjusted model (table 2).

Relationships with patients significantly moderated the relationship between bureaucratic demands and patient related-burnout ($b_{interactionterm}$ = –0.15; 95% CI, –0.27 to –0.04; p = 0.01). Specifically, bureaucratic demands were significantly positively associated with patient-related burnout when physicians reported low (-1SD; mean of 3.25) or average (mean of 4.00) ratings on the quality of their relationships with patients. Specifically, the association between bureaucratic demands and patient-related burnout was stronger when physicians' ratings of relationships with patients were low (b_{low} = 0.21; 95% CI, 0.11 to 0.31; t = 4.18) than when ratings were average (p < 0.001 and $b_{average}$ = 0.10; 95% CI, 0.02 to 0.18; t = 2.35; p = 0.02). When physicians' ratings of patient relationships were high, there was no association between bureaucratic demands and patient-related burnout (b_{high}= 0.02; 95% CI, -0.09 to 0.13; t = 0.34; p = 0.73).

For peer review only - http://bmjopen.bmj.com/site/about/guidelines.xhtml

DISCUSSION

Principal findings

This study on associations between job demands, job resources and patient-related burnout showed that physicians with high workloads and few development opportunities report higher levels of patient-related burnout. Those with positive patient relationships are less likely to experience such burnout, even in the presence of excessive bureaucracy.

Strengths and limitations of the study

This multicentre study used widely validated instruments to examine associations between job demands, job resources and patient-related burnout, which were selected based on needs assessment of practicing physicians' needs in alignment with the theoretical assumptions of the job demands and resources model. However, other job demands and resources also may be relevant, especially in other settings (e.g. non-Dutch systems and primary care). Furthermore, our findings may not be generalizable to other health care systems, although our findings align with previous findings of related research – on associations between job demands, resources and burnout – in diverse health care systems.[23 25 32-34] Nonetheless, intensified research on the role of physician characteristics – across diverse systems and settings – is needed to clarify whether and how findings on job demands, resources and patient-related burnout should be tailored to specific physician subgroups.

In our study, the study sample was characterized by a high response rate, inclusion of physicians from multiple specialties, and the sex distribution of the sample was consistent with national data.[35] As study participation was voluntary, subscale scores may be subject to self-selection bias. However, the observed associations of job demands and resources with patient-related burnout – the main focus of the current study – are in agreement with related findings

For peer review only - http://bmjopen.bmj.com/site/about/guidelines.xhtml

For peer review only

from diverse settings, as detailed below.[20 22 36] Furthermore, the cross-sectional study design precluded the assessment of causal associations. Previous research has identified longitudinal associations of job demands and resources with burnout,[37 38] which should be clarified for patient-related burnout in particular.

Comparison with other studies

Our results align with previous findings that job demands are associated with patient-related burnout in general.[15 20] We additionally showed that levels of patient-related burnout were lower among physicians who experienced positive relationships with patients. Physicians perceived positive relationships with patients when they, for example, perceived gratitude from their patients, or felt a strong personal connection with patients. These resources of physicians' work showed to keep physicians going, even in the face of excessive demands.[39] Indeed, physicians experiencing highly positive relationships with patients did not report exhaustion (i.e. patient-related burnout), even when exposed to excessive bureaucracy. On the other hand, physicians reporting less positive relationships with patients reported higher levels of patient-related burnout in the face of excessive bureaucracy. In other words, bureaucratic demands are less likely to be associated with patient-related burnout when physicians experience positive relationships with patients; this may indicate positive relationships to buffer the potentially negative impact of excessive bureaucracy on patient-related burnout.

These findings align with those of other research based on the job demands and resources model, which showed that particular resources buffer the negative impact of demands on well-being.[21] Specifically, previous research showed life satisfaction or work engagement are less likely to be impaired by job demands when job resource levels are high.[32 39] In the case of patient-related burnout, the negative impact of bureaucratic demands in particular may be buffered by positive relationships with patients. Bureaucratic demands represent the extent to which physicians perceive policies and procedures as burdensome, ineffective or unnecessary.[27] Bureaucratic

For peer review only - http://bmjopen.bmj.com/site/about/guidelines.xhtml

demands and the related concept of administrative burden have been shown to be associated with work-related burnout.[11]

Work-related burnout is also more likely in the presence of high workloads,[40-42] which aligns with our study findings showing associations between workloads and patient-related burnout. Alarmingly, these findings may indicate that workloads may exhaust physicians in their core task – caring for patients. Patient-related burnout may, on the other hand, be less likely in the presence of ample development opportunities, consistent with related research;[20] development opportunities have also been shown to benefit physicians' work engagement, another indicator of well-being.[43] Development opportunities stimulate physicians' senses of capability, mastery and skill, which may enhance their sense of clinical competence and prevent stress or exhaustion in the face of clinically or emotionally demanding situations in patient care.[44] Indeed, learning and professional updating in the context of continuous medical education (CME) have been associated with lower levels of stress and burnout.[44] Therefore, CME activities may be considered when aiming to address patient-related burnout in medical practice.

In this study, patient-related burnout was not affected by job resources involving participation in decision making, leaders' inspiration or supportive collegial relationships. This is consistent with previous research confirming the absence of associations between these resources and physicians' well-being (i.e. work engagement and work-related burnout).[43 45 46] The lack of association with collegial relationships is surprising, as collegial and peer support has shown the potential to help physicians deal with stress and threats to well-being.[39] Such support may not be cultivated fully in the professional medical context due to time limitations or physicians' personal barriers (e.g. apprehension about peers' views on their ability to cope).[47 48] Full cultivation of collegial support could contribute to a sense of reduced professional isolation and the reduction of physicians' exhaustion in providing patient care.[39 49] For example, collegial support could be fostered by debrief groups in which peers exchange stressful experiences and together reflect on coping with challenges of patient care, e.g. emotionally demanding patients.[50]

For peer review only - http://bmjopen.bmj.com/site/about/guidelines.xhtml

Implications for practice and research

Although physicians are generally at low risk of patient-related burnout, even low levels of such burnout may seriously threaten the sustainability of their practice – as manifested by low levels of commitment to work18 and job satisfaction.[15] Thus, each patient-related burnout risk factor should be prevented or resolved, which could be facilitated by optimising the balance between job demands and resources. This could be achieved by implementing managerial interventions such as worker health surveillance[51 52] or by adapting organizational structures that facilitate job crafting, i.e. proactive strategies in increasing job resources and decreasing hindering demands, both at the individual and team level.[53] Hospitals could furthermore consider how to reduce demands that interfere with physician–patient relationships, or invest in professional development programmes (e.g. CME) that facilitate physicians' learning and development of positive relationships with patients. Such relationship development could also be fostered by addressing system- and organisation-level barriers to physicians' delivery of compassionate care (e.g. inadequate time with patients, unsupportive leadership, inadequate support personnel and non-facilitative practice structures).[54] Furthermore, positive relationships with patients could be fostered by mindfulness-based communication programs; these programs enhanced physicians' dedicated and non-judgmental attention towards patients' thoughts and emotions, which showed to facilitate empathy towards patients.[55-57] Such programmes have also been shown to promote physicians' self-compassion and self-care in stressful practice environments, and may thus contribute to physicians' well-being and the prevention of (patient-related) burnout.[57]

Efforts to prevent patient-related burnout should include the creation of a healthy workplace with consideration of physicians' and patients' input, characterised (according to the work life model) by reasonable workloads, control over the practice environment, stimulating rewards, a supportive community, fair treatment of staff and professional values.[7 58] Care delivery according

For peer review only - http://bmjopen.bmj.com/site/about/guidelines.xhtml

to professional values may be complicated by physician burnout, as burned-out physicians are twice as likely to exhibit low levels of professionalism (i.e. low adherence to treatment guidelines, lack of professional integrity and low levels of empathy).[1] However, the effects of patient-related burnout on physicians' professionalism remain unclear. Personal-related burnout, i.e. the degree to which physicians' exhaustion in their personal life, is associated with physicians' patient-centered attitudes,[59] yet this association has not been studied for patient-related burnout specifically. Furthermore, burnout in general has been associated with suboptimal patient care quality (e.g. patient safety and satisfaction),[1-3] yet, future research should also clarify this for patient-related burnout in particular.

For peer review only

References

1. Panagioti M, Geraghty K, Johnson J, et al. Association between physician burnout and patient safety, professionalism, and patient satisfaction: a systematic review and meta-analysis. *JAMA Int Med* 2018;178(10):1317-30.
2. Hall LH, Johnson J, Watt I, et al. Healthcare staff wellbeing, burnout, and patient safety: A systematic review. *PLoS One* 2016;11(7):e0159015.
3. Salyers MP, Bonfils KA, Luther L, et al. The Relationship Between Professional Burnout and Quality and Safety in Healthcare: A Meta-Analysis. *J Gen Int Med* 2016:1-8.
4. Dyrbye LN, Varkey P, Boone SL, et al. Physician satisfaction and burnout at different career stages. *Mayo Clin Proc* 2013; 88(12): 1358-1367.
5. Shanafelt TD, Balch CM, Bechamps GJ, et al. Burnout and career satisfaction among American surgeons. *Ann Surg* 2009;250(3):463-71.
6. Prins JT, Gazendam-Donofrio SM, Tubben BJ, et al. Burnout in medical residents: a review. *Med Educ* 2007;41(8):788-800.
7. Montgomery A, Panagopoulou E, Esmail A, et al. Burnout in healthcare: the case for organisational change. *BMJ* 2019;366:l4774.
8. National Academies of Sciences. Taking action against clinician burnout: a systems approach to professional well-being. Washington DC: National Academies Press 2019.
9. West CP, Dyrbye LN, Erwin PJ, et al. Interventions to prevent and reduce physician burnout: a systematic review and meta-analysis. *Lancet* 2016
10. Panagioti M, Panagopoulou E, Bower P, et al. Controlled Interventions to Reduce Burnout in Physicians: A Systematic Review and Meta-analysis. *JAMA Int Med* 2016
11. Rao SK, Kimball AB, Lehrhoff SR, et al. The Impact of Administrative Burden on Academic Physicians: Results of a Hospital-Wide Physician Survey. *Acad Med* 2017;92(2):237-43. doi: 10.1097/acm.0000000000001461
12. Boerjan M, Bluyssen SJ, Bleichrodt RP, et al. Work-related health complaints in surgical residents and the influence of social support and job-related autonomy. *Med Educ* 2010;44(8):835-44.
13. Shanafelt TD. Enhancing meaning in work: A prescription for preventing physician burnout and promoting patient-centered care. *JAMA* 2009;302(12):1338-40. doi: 10.1001/jama.2009.1385
14. Chou L-P, Li C-Y, Hu SC. Job stress and burnout in hospital employees: comparisons of different medical professions in a regional hospital in Taiwan. *BMJ Open* 2014;4(2):e004185.
15. Wright JG, Khetani N, Stephens D. Burnout among faculty physicians in an academic health science centre. *Paediatrics & child health* 2011;16(7):409-13.
16. Kristensen TS, Borritz M, Villadsen E, et al. The Copenhagen Burnout Inventory: A new tool for the assessment of burnout. *Work & Stress* 2005;19(3):192-207.
17. Ratnakaran B, Prabhakaran A, Karunakaran V. Prevalence of burnout and its correlates among residents in a tertiary medical center in Kerala, India: A cross-sectional study. *J Postgr Med* 2016;62(3):157.
18. Messias E, Gathright MM, Freeman ES, et al. Differences in burnout prevalence between clinical professionals and biomedical scientists in an academic medical centre: a cross-sectional survey. *BMJ Open* 2019;9(2):bmjopen-2018-023506.
19. Borritz M, Rugulies R, Christensen K, et al. Burnout as a predictor of self-reported sickness absence among human service workers: prospective findings from three year follow up of the PUMA study. *Occ Env Med* 2006;63(2):98-106.

For peer review only - http://bmjopen.bmj.com/site/about/guidelines.xhtml

20. Ilić IM, Arandjelović MŽ, Jovanović JM, et al. Relationships of work-related psychosocial risks, stress, individual factors and burnout–Questionnaire survey among emergency physicians and nurses. *Med Pr* 2017;68(2):167-78.
21. Bakker AB, Hakanen JJ, Demerouti E, et al. Job resources boost work engagement, particularly when job demands are high. *J Educ Psych* 2007;99(2):274.
22. Bakker AB, Demerouti E. The job demands-resources model: State of the art. *J Manag Psych* 2007;22(3):309-28.
23. Ståhl ACF, Ståhl C, Smith P. Longitudinal association between psychological demands and burnout for employees experiencing a high versus a low degree of job resources. *BMC Public Health* 2018;18(1):915.
24. Teoh K, Hassard J, Cox T. Doctors' perceived working conditions and the quality of patient care: a systematic review. *Work & Stress* 2019;33(4):385-413. doi: 10.1080/02678373.2019.1598514
25. Bakker AB, Lieke L, Prins JT, et al. Applying the job demands–resources model to the work–home interface: A study among medical residents and their partners. *J Voc Beh* 2011;79(1):170-80.
26. Veldhoven Mv, Jonge Jd, Broersen S, et al. Specific relationships between psychosocial job conditions and job-related stress: A three-level analytic approach. *Work & Stress* 2002;16(3):207-28.
27. Borry EL. A new measure of red tape: Introducing the three-item red tape (TIRT) scale. *Int Publ Manag J* 2016;19(4):573-93.
28. Williams ES, Konrad TR, Linzer M, et al. Refining the measurement of physician job satisfaction: results from the Physician Worklife Survey. *Med Care* 1999:1140-54.
29. Schönrock-Adema J, Heijne-Penninga M, van Hell EA, et al. Necessary steps in factor analysis: enhancing validation studies of educational instruments. The PHEEM applied to clerks as an example. *Med Teach* 2009;31(6):e226-e32.
30. Cronbach LJ. Coefficient alpha and the internal structure of tests. *Psychometrika* 1951;16(3):297-334.
31. Hayes AF. Introduction to mediation, moderation, and conditional process analysis: A regression-based approach: Guilford Publications 2017.
32. Hakanen JJ, Bakker AB, Demerouti E. How dentists cope with their job demands and stay engaged: the moderating role of job resources. *Eur J Oral Sc* 2005;113(6):479-87. doi: 10.1111/j.1600-0722.2005.00250.x
33. Laschinger HKS, Grau AL, Finegan J, et al. Predictors of new graduate nurses' workplace well-being: Testing the job demands–resources model. *Health Care Man Rev* 2012;37(2):175-86.
34. Schmidt KH, Diestel S. Job demands and personal resources in their relations to indicators of job strain among nurses for older people. *J Adv Nurs* 2013;69(10):2185-95.
35. Van der Meulen L. Aantal werkzame specialisten per specialisme en uitstroom van Specialisten in de komende 20 jaar: Capaciteitsorgaan, 2019.
36. Bakker AB. An evidence-based model of work engagement. *Curr Dir Psych Sc* 2011;20(4):265-69.
37. Wang Y, Huang J, You X. Personal resources influence job demands, resources, and burnout: A one-year, three-wave longitudinal study. *Soc Beh Pers* 2016;44(2):247-58.
38. Hakanen JJ, Schaufeli WB, Ahola K. The Job Demands-Resources model: A three-year cross-lagged study of burnout, depression, commitment, and work engagement. *Work & Stress* 2008;22(3):224-41. doi: 10.1080/02678370802379432
39. Wallace JE, Lemaire J. On physician well being—you'll get by with a little help from your friends. *Soc Sc Med* 2007;64(12):2565-77.
40. Pastores SM, Kvetan V, Coopersmith CM, et al. Workforce, workload, and burnout among intensivists and advanced practice providers: a narrative review. *Crit Care Med* 2019;47(4):550-57.
41. Bragard I, Dupuis G, Fleet R. Quality of work life, burnout, and stress in emergency department physicians: a qualitative review. *Eur J Emerg Med* 2015;22(4):227-34.

For peer review only - http://bmjopen.bmj.com/site/about/guidelines.xhtml

42. West CP, Dyrbye LN, Shanafelt T. Physician burnout: contributors, consequences and solutions. *J Int Med* 2018;283(6):516-29.
43. Scheepers RA, Emke H, Epstein RM, et al. The impact of mindfulness-based interventions on doctors' well-being and performance: A systematic review. *Med Educ* 2020;54:138-149. doi: 10.1111/medu.14020
44. Kushnir T, Cohen AH, Kitai E. Continuing medical education and primary physicians' job stress, burnout and dissatisfaction. *Med Educ* 2000;34(6):430-36. doi: 10.1046/j.1365-2923.2000.00538.x
45. Verweij H, van der Heijden FMMA, van Hooff MLM, et al. The contribution of work characteristics, home characteristics and gender to burnout in medical residents. *Adv Health Sc Educ* 2017;22(4):803-18. doi: 10.1007/s10459-016-9710-9
46. Hariharan TS, Griffin B. A review of the factors related to burnout at the early-career stage of medicine. *Med Teach* 2019;41(12):1380-91.
47. Wallace JE, Lemaire JB, Ghali WA. Physician wellness: a missing quality indicator. *Lancet* 2009;374(9702):1714-21.
48. Edwards N, Kornacki MJ, Silversin J. Unhappy doctors: what are the causes and what can be done? *BMJ* 2002;324(7341):835.
49. Shapiro J, Galowitz P. Peer support for clinicians: A programmatic approach. *Acad Med* 2016;91(9):1200-04.
50. Lightowlers SV, Thompson MK, Hunt SL. Not all interventions for burnout cost money: the value of debrief groups. *BMJ* 2017;358:j4377. doi: 10.1136/bmj.j4377
51. Ruitenburg MM, Plat M-CJ, Frings-Dresen MH, et al. Feasibility and acceptability of a workers' health surveillance program for hospital physicians. 2015;28(4):731.
52. Ruitenburg MM, Frings-Dresen MHW, Sluiter JK. How to define the content of a job-specific worker's health surveillance for hospital physicians? *Safe Health Work* 2016;7(1):18-31. doi: http://dx.doi.org/10.1016/j.shaw.2015.08.004
53. Hakanen JJ, Peeters MC, Schaufeli WB. Different types of employee well-being across time and their relationships with job crafting. *J Occ Health Psych* 2018;23(2):289.
54. Rider EA, Gilligan MC, Osterberg LG, et al. Healthcare at the Crossroads: The Need to Shape an Organizational Culture of Humanistic Teaching and Practice. *J Gen Int Med* 2018;33(7):1092-99. doi: 10.1007/s11606-018-4470-2
55. Verweij H, van Ravesteijn H, van Hooff ML, et al. Does mindfulness training enhance the professional development of residents? A qualitative study. *Acad Med* 2018;93(9):1335-40.
56. Beckman HB, Wendland M, Mooney C, et al. The impact of a program in mindful communication on primary care physicians. *Acad Med* 2012;87(6):815-19.
57. Scheepers RA, Emke H, Epstein RM, et al. The impact of mindfulness-based interventions on doctors' well-being and performance: A systematic review. *Med Educ* 2020;54:138-149. doi: 10.1111/medu.14020
58. Leiter MP, Maslach C. Areas of worklife: A structured approach to organizational predictors of job burnout. Emotional and physiological processes and positive intervention strategies: Emerald Group Publishing Limited 2003:91-134.
59. Ahmad W, Ashraf H, Talat A, et al. Association of burnout with doctor–patient relationship and common stressors among postgraduate trainees and house officers in Lahore—a cross-sectional study. *PeerJ* 2018;6:e5519.

For peer review only - http://bmjopen.bmj.com/site/about/guidelines.xhtml

Table 1. Characteristics of the study population

Characteristics	N (%)
Number of respondents	465 (100)
Male	222 (47.7)
Female	243 (52.3)
Type of respondent	
Medical specialist	385 (82.8)
Resident	80 (17.2)
Specialty	
Surgical	193 (41.5)
Non-surgical	226 (48.6)
Supporting	32 (6.9)
Non-medical	14 (3.0)
Years after completing M.D.	
0-5	40 (8.6)
6-10	93 (20.0)
11-15	88 (18.9)
16-21	80 (17.2)
22-45	161 (34.6)
46+	3 (0.6)
Type of contract	
Full-time	257 (55.3)
Part-time	208 (44.7)

For peer review only

For peer review only - http://bmjopen.bmj.com/site/about/guidelines.xhtml

Table 2. Unadjusted and adjusted models predicting the effect of job resources and job demands on patient related burnout by using total mean scores (model 1) or subscale scores (model 2)

		Unadjusted model	Adjusted model#
		Regression coefficient (95% CI; p-value)	Regression coefficient (95% CI; p-value)
Job resources	Total mean score	-1.15 (-0.28 to -0.01; 0.03)	-0.17 (-0.31 to -0.04; 0.01)
	Relationships with colleagues	0.09 (-0.04 to 0.22; 0.17)	0.08 (-0.06 to 0.21; 0.26)
	Participation in decision making	0.02 (-0.07 to 0.11; 0.73)	0.02 (-0.08 to 0.11; 0.72)
	Development opportunities	-0.17 (-0.26 to -0.07; 0.00)	-0.18 (-0.27 to -0.08; 0.00)
	Leaders' inspiration	0.04 (-0.04 to 0.12; 0.30)	0.03 (-0.05 to 0.11; 0.41)
	Relationships with patients	-0.12 (-0.22 to -0.03; 0.01)	-0.12 (-0.22 to -0.03; 0.01)
Job demands	Total mean score	0.29 (0.17 to 0.42; 0.00)	0.29 (0.17 to 0.42; 0.00)
	Workload	0.34 (0.23 to 0.45; 0.00)	0.36 (0.25 to 0.48; 0.00)
	Bureaucratic demands	0.09 (0.01 to 0.17; 0.03)	0.08 (-0.00 to 0.16; 0.06)

#Adjusted for: physicians' sex, years of experience after obtaining an M.D., type of contract (full-time/part-time), and type of respondent (medical specialist/resident).

For peer review only

Supplementary tables

Table A1. The job resources, job demands and patient-related burnout items and corresponding psychometric properties

Construct (Cronbach's α)	Subscale (Cronbach's α)	Mean (SD)*	Item code (corrected item-total correlation)	Factor loadings	Item
Job resources	Relationships with colleagues (α = 0.75)	3.43 (0.47)	(0.34)	0.35	Asking colleagues for support
			(0.62)	0.71	Good understanding with colleagues
			(0.54)	0.60	Conflicts with colleagues
			(0.65)	0.77	Pleasant atmosphere between colleagues
			(0.48)	0.56	Unpleasant events with colleagues
	Participation in decision-making (α = 0.82)	3.60 (0.74)	(0.63)	0.63	Participation in important decisions
			(0.73)	0.85	Participation in assignment of tasks
			(0.60)	0.68	Participation in timetable planning
			(0.61)	0.63	Influence on work
	Development oppurtunities (α = 0.84)	4.14 (0.69)	(0.68)	-0,82	Opportunity to learn new things in work
			(0.75)	-0.77	Opportunity to grow and develop
			(0.68)	-0.72	Opportunity to achieve something in work
	Leaders' inspiration (α = 0.91)	2.64 (0.82)	(0.83)	0.87	Enthusiasm of the leader
			(0.78)	0.83	Good example of the leader
			(0.82)	0.85	Ratification of the leader
			(0.79)	0.81	Clear vision of the leader
	Relationships with patients (α = 0.71)	3.81 (0.62)	(0.33)	0.29	I find my present clinical work personally rewarding
			(0.61)	0.72	I feel a strong personal connection with my patients
			(0.57)	0.74	The gratitude displayed by my patients keeps me going
			(0.51)	0.64	I am having a positive impact on a socio-economically disadvantaged population
Job demands	Bureaucratic load (α = 0.74)	3.11 (0.73)	(0.52)	0.60	Not burdensome to burdensome policies and procedures

For peer review only

			(0.58)	0.72	Necessary to unnecessary policies and procedures
			(0.62)	0.80	Effective to ineffective policies and procedures
	Workload (α = 0.79)	2.85 (0.52)	(0.39)	0.47	Amount of work
			(0.58)	0.69	Extra work to complete tasks
			(0.61)	0.70	Hurry in work
			(0.56)	0.64	Arrear in work
			(0.54)	0.59	Problems with work pace
			(0.60)	0.65	Problems with workload
Patient-related burnout	Patient-related burnout (α = 0.83)	1.91 (0.63)	(0.65)	0.81	Do you find it hard to work with patients?
			(0.67)	0.69	Do you find it frustrating to work with patients?
			(0.72)	0.56	Does it drain your energy to work with patients?
			(0.61)	0.50	Do you feel that you give more than you get back when you work with patients?
			(0.54)	0.74	Are you tired of working with patients?
			(0.46)	0.75	Do you sometimes wonder how long you will be able to continue working with patients?

*SD = standard deviation

For peer review only

Table A2. Inter-scale correlations for the subscales of the job resources and job demands constructs

	**Job resources**					**Job demands**	
	Relationships with colleagues	Participation in decision-making	Development possibilities	Inspiration by the leader	Relationships with patients	Bureaucratic load	Workload
Relationships with colleagues	1	0.32**	0.30**	0.34**	0.15**	-	-
Participation in decision making	-	1	0.40**	0.38**	0.17**	-	-
Development oppurtunities	-	-	1	0.34**	0.28**	-	-
Leaders' inspiration	-	-	-	1	0.10*	-	-
Relationships with patients	-	-	-	-	1	-	-
Bureaucratic load	-	-	-	-	-	1	0.15**
Workload	-	-	-	-	-	-	1

*significant Pearson correlation p<0.01
** significant Pearson correlation p<0.05

For peer review only

STROBE Statement—checklist of items that should be included in reports of observational studies

	Item No	Recommendation	Page number
**Title and abstract**	1	(*a*) Indicate the study's design with a commonly used term in the title or the abstract	1
		(*b*) Provide in the abstract an informative and balanced summary of what was done and what was found	2
**Introduction**			
Background/rationale	2	Explain the scientific background and rationale for the investigation being reported	6-7
Objectives	3	State specific objectives, including any prespecified hypotheses	7
**Methods**			
Study design	4	Present key elements of study design early in the paper	7
Setting	5	Describe the setting, locations, and relevant dates, including periods of recruitment, exposure, follow-up, and data collection	7
Participants	6	(*a*) *Cohort study*—Give the eligibility criteria, and the sources and methods of selection of participants. Describe methods of follow-up<br>*Case-control study*—Give the eligibility criteria, and the sources and methods of case ascertainment and control selection. Give the rationale for the choice of cases and controls<br>*Cross-sectional study*—Give the eligibility criteria, and the sources and methods of selection of participants	7-8
		(*b*) *Cohort study*—For matched studies, give matching criteria and number of exposed and unexposed<br>*Case-control study*—For matched studies, give matching criteria and the number of controls per case	
Variables	7	Clearly define all outcomes, exposures, predictors, potential confounders, and effect modifiers. Give diagnostic criteria, if applicable	8-9
Data sources/ measurement	8*	For each variable of interest, give sources of data and details of methods of assessment (measurement). Describe comparability of assessment methods if there is more than one group	8-9
Bias	9	Describe any efforts to address potential sources of bias	9-10
Study size	10	Explain how the study size was arrived at	7
Quantitative variables	11	Explain how quantitative variables were handled in the analyses. If applicable, describe which groupings were chosen and why	9-10
Statistical methods	12	(*a*) Describe all statistical methods, including those used to control for confounding	9-10
		(*b*) Describe any methods used to examine subgroups and interactions	9-10
		(*c*) Explain how missing data were addressed	NA
		(*d*) *Cohort study*—If applicable, explain how loss to follow-up was addressed<br>*Case-control study*—If applicable, explain how matching of cases and controls was addressed<br>*Cross-sectional study*—If applicable, describe analytical methods taking account of sampling strategy	NA
		(*e*) Describe any sensitivity analyses	NA

For peer review only - http://bmjopen.bmj.com/site/about/guidelines.xhtml

Continued on next page

**Results**			
Participants	13*	(a) Report numbers of individuals at each stage of study—eg numbers potentially eligible, examined for eligibility, confirmed eligible, included in the study, completing follow-up, and analysed	11
		(b) Give reasons for non-participation at each stage	NA
		(c) Consider use of a flow diagram	NA
Descriptive data	14*	(a) Give characteristics of study participants (eg demographic, clinical, social) and information on exposures and potential confounders	Table 1
		(b) Indicate number of participants with missing data for each variable of interest	NA
		(c) *Cohort study*—Summarise follow-up time (eg, average and total amount)	
Outcome data	15*	*Cohort study*—Report numbers of outcome events or summary measures over time	
		Case-control study—Report numbers in each exposure category, or summary measures of exposure	
		Cross-sectional study—Report numbers of outcome events or summary measures	Table A1
Main results	16	(*a*) Give unadjusted estimates and, if applicable, confounder-adjusted estimates and their precision (eg, 95% confidence interval). Make clear which confounders were adjusted for and why they were included	Table 2
		(*b*) Report category boundaries when continuous variables were categorized	NA
		(*c*) If relevant, consider translating estimates of relative risk into absolute risk for a meaningful time period	NA
Other analyses	17	Report other analyses done—eg analyses of subgroups and interactions, and sensitivity analyses	11
**Discussion**			
Key results	18	Summarise key results with reference to study objectives	12
Limitations	19	Discuss limitations of the study, taking into account sources of potential bias or imprecision. Discuss both direction and magnitude of any potential bias	12-13
Interpretation	20	Give a cautious overall interpretation of results considering objectives, limitations, multiplicity of analyses, results from similar studies, and other relevant evidence	13-14
Generalisability	21	Discuss the generalisability (external validity) of the study results	12
**Other information**			
Funding	22	Give the source of funding and the role of the funders for the present study and, if applicable, for the original study on which the present article is based	4

*Give information separately for cases and controls in case-control studies and, if applicable, for exposed and unexposed groups in cohort and cross-sectional studies.

Note: An Explanation and Elaboration article discusses each checklist item and gives methodological background and published examples of transparent reporting. The STROBE checklist is best used in conjunction with this article (freely available on the Web sites of PLoS Medicine at http://www.plosmedicine.org/, Annals of Internal Medicine at http://www.annals.org/, and Epidemiology at http://www.epidem.com/). Information on the STROBE Initiative is available at www.strobe-statement.org.

For peer review only
